# Efficiency of a pilot scheme for the separate collection of the biowaste from municipal solid waste in Spain

**DOI:** 10.1038/s41598-021-90957-2

**Published:** 2021-06-02

**Authors:** Antonio Gallardo, Francisco J. Colomer-Mendoza, Mar Carlos-Alberola, Cristóbal Badenes, Natalia Edo-Alcón, Joan Esteban-Altabella

**Affiliations:** Department of Mechanical Engineering and Construction, Universitat Jaume I, Ave. Vicent Sos Baynat, s/n, 12071 Castellón, Spain

**Keywords:** Environmental sciences, Environmental social sciences

## Abstract

According to EU regulations, member states shall take measures to encourage the recycling of biowaste in a way that fulfils a high level of environmental protection. In Spain, the separate collection of biowaste is only implemented in some regions. For this reason, a pilot scheme based on an information campaign and the location of a specific brown container for biowaste in specific zones of the city was carried out in Castelló de la Plana (Spain) over a period of six months. In this period, the collection and composition of the biowaste was monitored in depth with the goal of determining the evolution of the efficiency of the new collection system over time. In the zones, the quality rate in the biowaste container increased as the pilot study progressed, finally reaching 90%. The rate of biowaste separation also increased in the three zones over time, although in different ways, which means that there is greater collaboration on the part of citizens. On the other hand, an analysis of the rate of net biowaste daily collection from zones 2 and 3 has shown that their value increases as the rate of containerization of biowaste decreases. Therefore, to obtain better results it will be necessary to increase the containerization of biowaste, that is, to reduce the distance from the citizen to the container. It can thus be said that there is a positive evolution of the experience, which boosts confidence when it comes to implementing the system throughout the city.

## Introduction

The European Union (EU) promotes a new plan of the waste management focused on the principles of the Circular Economy, attending to the Circular Economy Package of the EU in terms of Directives 2018/849, 2018/850, 2018/851 and 2018/852, signed on 30 May 2018.

Therefore, the EU Member States will have to achieve the separation of biowaste, which will contribute to the protection of the environment^1,2^. In addition, important benefits would be obtained such as the reduction of greenhouse gas emissions, generation of biogas and production of good quality compost. This will contribute to the improvement of the quality of the soil, the use and efficiency of resources and energy self-sufficiency^3^.

In Spain, the regulatory framework comprises the Waste and Polluted Soil law 11/2011 and the Waste Framework National Plan 2016–2022 states that the regional authorities must promote measures to collect biowaste separately and the aim is that before 2020 the amount of MSW to be reused or recycled (paper-cardboard, glass, metals, plastics, biowaste and other recyclable fractions) must reach, as a whole, 50% in weight. Finally, in 2019 the Valencian Community, the Spanish region where Castelló de la Plana is located, published its Integrated Solid Waste Plan (Decree 55/2019, 5 April 2019) that incorporates a mandatory separate biowaste collection in towns with more than five thousand inhabitants.

Moreover, separate collection of biodegradable waste provides a better-quality raw material for the production of fertilisers than material obtained from the mechanical separation of municipal solid waste^4^ and could be considered the best solution for reducing the global warming potential and for improving the useful life of the sanitary landfill^5^.

The main threat of the mismanagement of the biowaste is the production of methane by anaerobic fermentation in landfills. These emissions accounted for 3% of total greenhouse gas emissions in the EU-15 in 1995. The second threat, especially from municipal biological waste, is the generation of leachate, which can be an important source of contamination if it is not controlled^6^. In Ireland, for example, it is not allowed to receive wastes in landfills if they have not been previously treated according to the standards of the Irish Environmental Protection Agency^6^. Moreover, Member States shall ensure that, by 31 December 2023, biowaste is either separated and recycled at source, or is collected separately and is not mixed with other types of waste. In fact, works carried out in Catalonia (Spain) with the life cycle assessment demonstrated that the worst option for biowaste management is the traditional landfill^7^.

Generally speaking, the proportion of biowaste tends to be higher in developing countries than in developed countries. According to works carried out in developed countries, the proportion of biowaste is lower around 28.2% in Marche Region, Adriatic Sea side, Italy^8^, 35% in Greece^9^, 30% in London, Canada^10^, 29.2% in Latvia^11^, or 34% as average in European Union^12^. However, in developing countries the proportion of biowaste is significantly higher, according to some works, such as in Perhentian Island in southern China, the proportion of biowaste in MSW was 71.73%^13^, in Kathmandu (Nepal) 71%^14^, 72.12% in Lahore (Pakistan)^15^, 66% in Siem Reap, Cambodia^16^, or 64% in Kumasi, Ghana^10^. However, there are studies in which the proportion of biowaste varies inexplicably from one city to another within the same region of a country,such is the case of five cities in southern Sweden with values of 34.0%, 48.2%, 47.7%, and 46.7%^17^.

In some German cities, separate collection of household biowaste affects the quality and final composition of the recovered materials. In fact, when the biowaste was separated, the rest of the waste was reduced by up to 30%. Furthermore, the residual waste had less humidity, which improves the efficiency of the incineration plants^18^. In the food industry, the efficient separation of biowaste is even more important due to the large quantities of waste that is produced. Separation at source in the industries and factories is key to improve the quality of the resources obtained from biowaste. In addition, the separation of biowaste is a key action in the circular economy. As reported in literature sources creating a circular bioeconomy based on effective use of biomass (including biowaste) is one of the major global concerns. Biomass and biowastes are highly significant in a circular economy in terms of material products and the provision of energy^19^, since biowaste separate collection and its treatment play an important role in meeting the requirements of the circular economy has been confirmed^20^. Biowastes, annually generated at millions of tonnes scale worldwide, must enter a value chain crucial to rethink the planetary welfare in terms of circular economy, where the concept of sustainable growth has to be implemented through a closed loop for the recycling of any material or its transformation into other resources without harming and/or depleting the natural ecosystem^21^. Therefore, on the one hand, education and awareness of society and companies is essential, and on the other hand, investment in support facilities^22^.

Sometimes, when separation is done properly, investments in source separation are very profitable. As an example, in Southampton (UK), separate collection of biowaste could save the council £690,000 each year, despite having to incur a significant cost on vehicle adaptation and construction of the transfer stations^23^. In two UK cities (Cardiff and Southampton) a survey of 100 people on recycling, awareness and waste separation was carried out. In areas where selective collection of food was carried out (Cardiff), recycling rates and citizen satisfaction were higher. In the area with no separate collection (Southampton) over 75% of respondents said they would like to have a separate collection system and would participate if available^23^. In Portugal, a comparative study was carried out on the costs of separate collection. It was concluded that the cases of separate collection of biowaste did not imply an overall increase in costs in the service, they could even decrease them if more than 40% of the population (threshold for the case study) participate in the system^24^.

On the other hand, the collection system, the levels of separation at source, the urban density of the village and the obligation to use compostable bags are factors which influence to reduce the percentage of inappropriate material in the biowaste fraction^25^.

In Spanish cities, following the EU regulations, separate collection of biowaste is being applied. Taking into account the European standards and the different fractions that can be separated, there are eight selective collection systems^26^ like the five-containers model: glass, paper-cardboard, light packaging, biowaste and reject, although with little implementation.

This paper presents the results obtained in a pilot project for the source-separation collection of biowaste applied to the city of Castelló de la Plana (in eastern Spain). To this end, a methodology for the development of the pilot project is proposed. Additionally, the degree of the efficiency of the biowaste source-separation was determined in order to design the model to be implemented throughout the city. For this purpose, first of all, a set of indicators were defined. Second, details of the pilot experiment are given. The results of the experiment were treated statistically in order to organize the information. Both, the waste from the mixed container and that from the new biowaste container, were characterized to determine the exact waste composition and subsequently the data were compared. From all this work, conclusions about the implementation of a new biowaste container in a town were then extracted.

## Methodology

The study was divided into five stages: (i) objectives, indicators and scope of the study, (ii) identification of the study area, (iii) definition and dissemination of an information and awareness campaign (information campaign), (iv) experimental design, and (v) data analysis.

### Objectives, indicators and scope of the study

The aim of the pilot study is to determine the degree of the efficiency of the biowaste separate collection system over time and how the type of separate collection affects it. For this purpose, the following specific objectives have been proposed:

- Determine the degree of the efficiency of the biowaste collection system.
- Determine the degree of the variation in the efficiency over time.
- Determine whether the selective collection model can influence the degree of efficiency of the biowaste collection system.
- Determine the variation in the composition of the mixed waste (mx) container with or without selective collection of the biowaste fraction.

In order to determine the degree of efficiency of a collection system, first of all, it is necessary to define a number of indicators. In this work, efficiency is defined in terms of the extent to which clean materials are recovered at source, that is to say, materials deposited in containers. It is expressed a set of indicators defined by^27^ such as the separation rate (SR), the net separation rate (NSR) and the quality in container rate (QCR) shown in Eqs. (1), (2) and (3).

1 $${SR}_{i} (\%)=100\cdot \frac{amount\; of \; waste \; collected \; in \; container \; for \; i}{total \;amount\; of \; i \; waste \; generated}$$

For example, for i = biowaste (bw), SR_bw_ is the gross amount of biowaste (biowaste and inappropriate materials) collected in a container for biowaste in respect of the total biowaste generated, expressed in percentage.

2 $${NSR}_{i} (\%)=100\bullet \frac{amount \; of \; i \; waste \; collected \; in \; container \; for \; i }{total \; amount \; of \; i \; waste \; generated}$$

For example, for biowaste, NSR_bw_ is the amount of net biowaste collected in a container for biowaste in respect of the total biowaste generated, expressed in percentage.

3 $${QCR}_{i} (\%)=100\bullet \frac{amount \; of \; waste \; collected \; correctly \; in \; container \; for \; i }{total \; amount \; of \; waste \; collected \; in \; container \; for \; i}$$

For example, for biowaste, QCR_bw_ is the amount of net biowaste collected in a container for biowaste in respect of the gross biowaste (biowaste and inappropriate materials) dumped in the container, expressed in percentage.

The SR_i_ and QCR_i_ indicators are two useful indicators to know the number of inappropriate materials in the container. In the case of separate collection of the biowaste, the inappropriate material consists of plastic, glass, paper-cardboard, brick, etc. In order to compare the collection in different scenarios, the daily collection rate (DCR) and the containerization rate (CR) were defined (Eqs. 4 and 5):

4 $${DCR}_{i} (kg/in\cdot day)=\frac{amount \; of \; i \; waste \; collected \; in \; one \; day \; in \; an \; area }{inhabitant \; in \; this \; area}$$

For example, for biowaste, DCR_bw_ is the amount of biowaste collected per inhabitant and day.

5 $$CR (inh/container)=\frac{inhabitants}{containers}$$

The scope of the study has been focused only on the previous mixed waste fraction and the new biowaste fraction. The pilot study lasted six months, from 23 January to 20 July 2017. Previously, an information and awareness campaign for citizens was carried out, which lasted three months.

### Identification of the study area

Castelló de la Plana is a coastal city on the Mediterranean Sea (39.9857° N 0.0494° W), located in the Valencian Community in eastern Spain, with a population of 170,990 inhabitants in 2016 and a Mediterranean climate. Currently, in this city, people separate the waste into four fractions: mixed waste (biowaste and reject), paper/cardboard, light-packaging (beverage cartons, plastic and cans) and glass.

The mixed waste fraction is collected by means of three kerbside systems: (i) mechanical back-loading truck and 340 L containers. The distance between containers is 20–30 m; (ii) automatic side-loading truck and 1,100 L containers. The distance between containers is 50–60 m, and (iii) automatic side-loading truck and 3,200 L containers. The distance between containers is 100–120 m. Citizens deposit the glass fraction, paper/cardboard fraction and light-packaging fraction in drop-off areas.

The frequency of the collection of mixed waste (mx) is six days a week. The collection of mixed waste in 2016 was 56,875 tons, which represents a DCR_mx_ of 0.91 kg/inh·day.

Nowadays, the mixed waste is carried to a mechanical biological treatment plant where the recyclable materials are recovered (paper-cardboard, light-packaging and glass) and where the bio-stabilized is elaborated (low quality compost). The reject fraction is carried to a landfill. An extension of the plant and a new line to treat exclusively the biowaste collected separately are projected for 2021. The results of this work will be useful to design this new treatment line.

To carry out the pilot study, it was decided to define three study areas, one for each kerbside system. Table 1 shows the characteristics of each area. The zones have different numbers of inhabitants and containers.

**Table 1:** Characteristics of the study areas.

Zone	Inhabitants	Collection system	Containers		
			Vol. (L)	No. mixed waste	No. biowaste
Zone 1 (city centre)	3956	Mechanical back-loading truck	340	72	40
Zone 2 (north of the city)	1451	Mechanical back-loading truck	1100	18	10
Zone 3 (west city)	2244	Automatic side-loading truck	3200	8	6

Zone 1 is located in the city centre, which corresponds to the old city. It is an area where a low-density residential area is combined with commercial and restaurant areas. Zone 2 is located in the northern district of the city. It corresponds to a wide area with a high-density residential area with several green areas and little commerce. Zone 3 is located in the west of the city and has similar characteristics to Zone 2. Finally, Zone 4 was also defined, close to Zone 2, where the selective collection of biowaste was not implemented. Samples were taken in this area to determine the composition of the mixed waste container of the current MSW collection system in Castelló de la Plana.

### Information campaign

**Figure 1:**
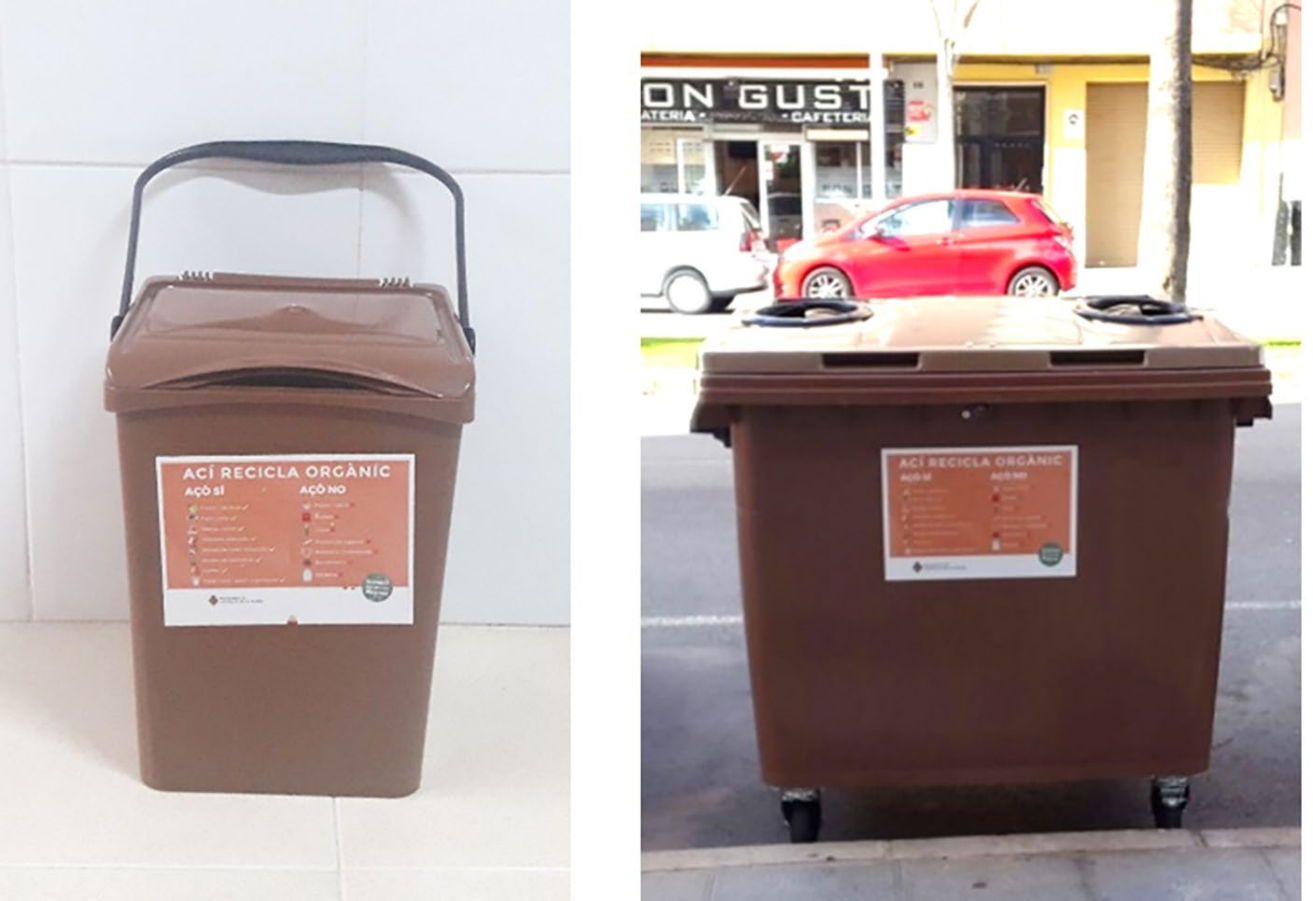
10 L bin (left) and 1,100 L container (right).

Before starting the pilot study, an information and awareness campaign was carried out in Zone 1, Zone 2 and Zone 3. This consisted in informative talks given in neighbours’ associations about the pilot scheme, its objective, the environmental benefits of its implementation and the importance of their participation. The inhabitants were provided with a 10 L brown plastic bin (Fig. 1 left) and biodegradable bags to separate their biowaste. They were also informed about the types of waste that they should deposit in that brown bin and in which container they should finally deposit the biowaste bags (brown container in the drop-off areas, Fig. 1 right). Both the brown bin and the brown container have an identification sticker indicating the material to be deposited. The mixed waste container in the drop-off areas is actually green, so there is a clear difference.

### Experimental design

The experiment consisted in defining the number, the adequate volume and location of the biowaste containers in each zone (Zone 1, Zone 2 and Zone 3) and their collection. The decision was made to install containers with the same volume as the mixed waste containers but with a different colour (brown), the distinctive colour of the corresponding bins that is reminiscent of food and garden waste (i.e. biowaste). Brown is the colour that is used exclusively for biowaste collection throughout Europe as opposed to the different colours that may be used for the bins for the other components^28^. The number of biowaste containers was lower than the number of mixed waste containers due to economic reasons. Table 1 shows the number of biowaste containers located in each area.

Containers were collected three times a week, since a large volume of biowaste was not expected. Each zone had an independent collection route. Once the collection was complete, the collection truck was weighed at the treatment plant, so daily collection data were available for the three areas. In Zone 1, due to different technical reasons, collection began 6 weeks later than in the rest.

Sampling and laboratory characterization were then scheduled to determine the average composition of the biowaste in the brown container in each of the three areas. Sampling and characterization were also scheduled to determine the composition of the mixed waste container in zone 4. To calculate the number of samples needed and their size, the “Methodology developed by the European Commission in 2004 for the Analysis of Solid Waste (SWA-Tool)” was used.

In order to determine the composition, the waste was separated into 13 categories: biowaste (food and garden waste), metal packaging (cans, etc.), other metals, clean paper/cardboard, dirty paper/cardboard, plastic packaging (food and beverage packaging, plastic bags, etc.), other plastics, sanitary cellulose, beverage carton packages, textile, glass packaging, others (flat glass, rubber and leather, wood, hazardous waste, electric and electronic wastes and inert) and fines (material less than 10 mm).

After the experimental stage, the results obtained in the four zones were analysed.

### Data analysis

In order to know the sample size, the SWA-Tool methodology was used. The number of samples was determined using Eq. (6).

6 $$n={\left(\frac{{t}_{\alpha ;n-1}\cdot VC}{\varepsilon }\right)}^{2}$$

where: n is the number of samples required, t_α;n−1_ is the deviation from the accepted mean value to achieve the desired confidence level (α − 1), for the “t” distribution. VC (variation coefficient) is the variance that we hope to find in the population (expressed as a decimal). ɛ is the maximum margin of error (expressed as a decimal).

To calculate VC, it is necessary to know previously the data about the mean and the standard deviation (st. dev.) of the waste composition.

Several statistical tests were carried out to evaluate the differences between datasets using the free software R commander^©^. The tests used were the Shapiro–Wilk test (test for normal distribution) to decide which statistical test to use, the Levene test (test for homogeneity of variances), ANOVA to compare the means and to verify differences among several tests, the Kruskal–Wallis test to compare two samples (used when data are not normally distributed) and finally, the Dunn post-hoc test that was used to perform multiple comparisons by pairs to identify the means that were different.

## Results and discussion

### Size and number of samples

To calculate the minimum number of samples needed to determine the composition of biowaste and mixed waste in the containers, the chosen confidence level was 95%, so the value ${t}_{\alpha ;n-1}$= 1.960. The means and standard deviations were obtained from previous characterizations carried out by the Castelló de la Plana City Council. For the calculation, only the biowaste fraction has been considered, since it is the one of interest for the study. Finally, a margin of error of 10% has been assumed.

Table 2 shows the data needed to perform the calculation and the results obtained after applying Eq. (1).

**Table 2:** Sample number calculation.

Container	Waste fraction	Earlier data			${t}_{0.05;\infty }$	$\varepsilon$	n
		Mean (%)	St. dev (%)	VC (a decimal)			
Biowaste	Biowaste	77.02	10.12	0.13	1.96	0.1	7
Mixed waste	Biowaste	52.55	4.46	0.08	1.96	0.1	3

According to Table 2 and Eq. (1), it will be necessary to take at least seven samples in each of the three zones to determine the biowaste fraction with a confidence level of 95% and an error of 10%. In the case of the mixed waste container, only three samples will be necessary in each of the four areas.

One of the objectives of the study was to determine the variation in the composition of the biowaste container over the duration of the experiment, so it was decided to increase the number of samples and characterize 22 samples in Zone 1, 27 samples in Zone 2 and 25 samples in Zone 3.

In the case of the biowaste container, the samples were evenly distributed over the six months of the pilot study. In the case of the mixed waste container, the sampling was concentrated in the last four months in the four areas.

Regarding the minimum sample size required, the SWA-Tool recommends that it should be equal to the volume of a container similar to those existing in the study area, without taking into account the amount of waste contained within it. It also establishes that if there are containers of different sizes in the same study area, the volume of the most commonly used type of container should be chosen as the sample size. Therefore, the chosen sample sizes correspond to the volume of the existing containers in each area. For Zone 4, the chosen volume was 1,100 L.

### Quality container rate in biowaste collection and variation over time

One of the indicators of the efficiency of the biowaste (bw) collection system is the Quality in Container Rate (QCR_bw_). The percentage of biowaste in the container coincides with this indicator.

The average composition for each of the three zones is shown in Table 4. It can be seen that in the three areas, the percentage of biowaste is high, between 79.75 and 82.74%, although there is still around 20% of inappropriate, which will end up being a reject in the treatment plant. However, the removal of impurities is still a challenge, due to the high heterogeneity of the biowastes and their varying compositions according to seasons and origins^29^. The estimates demonstrate that the collection system, the global levels of separate collection, the urban density of the municipality and the requirement to use compostable bags may be the main drivers of impurity levels in biowaste^25^.

These QCR_bw_ values are lower than those obtained in an experiment carried out in the city of Reggio Calabria (Italy), where the biowaste bins received 89% of biowaste, after a public awareness campaign. But in this case, the separate collection of biowaste was door-to-door and had been in place for several years^28^. However, the values are similar to those recorded in Catalonia (Spain), where the majority of characterizations of the biowaste fraction contained between 10 and 20% of inappropriate material. Nevertheless, in some cities this figure was as high as 40% or 50%^30^. On the other hand, in an experience experiment with only 425 inhabitants, Boelens et al.^31^ obtained very low inappropriate materials values of around 3% in Antwerp (Belgium) (Table 3).

**Table 3:** Quality in container rate of biowaste in several regions around Europe.

	QCR_bw_(%) Biowaste container
Castelló de la Plana (Spain) Zone 1	79.75
Castelló de la Plana (Spain) Zone 2	82.74
Castelló de la Plana (Spain) Zone 3	81.18
Catalonia (Spain)^30^^a^	80—90
Antwerp (Belgium)^31^	97
Regio Calabria (Italy)^28^	89
Ústí nad Labem (Czech Republic)^41^	70—90
Catalonia (Spain)^4^	89.3
Catalonia (Spain)^44^	90

^a^In some cities in Catalonia QCR_bw_ was 50–60%.

The fraction of “fines” is the most abundant in the inappropriate part. This is a fraction with materials less than 10 mm in size, such as dirt, dust, stones, microplastics, metals, etc. Secondly, there is plastic packaging, consisting of bags, bottles, dirty food packaging, etc. The amount of non-biodegradable bags that appeared could have been lower if a greater number of biodegradable bags had been distributed to citizens. In fact, the use of modern compostable bags is starting to be implemented in some European countries and encourages separation of biowaste at source. These compostable bags are made of biodegradable polymers, often from renewable sources^32^. Furthermore, in a region of Spain (Catalonia) a pilot project has been carried out since 1996 on separate collection of biowaste. Biodegradable waste from homes, shops, markets, restaurants, etc. are collected using a door-to-door system. Currently, this system covers 95% of the population with high participation, also due to the previous distribution of bins and biodegradable bags to citizens. Biowaste is treated by means of a combined anaerobic digestion and composting systems (Urban^33^. In other works, the relationship between the separate collection system and the quality of the biowaste was verified. The door-to-door collection system was the one with the highest quality of biowaste^30^.

According to environmental impacts, works carried out by Iriarte et al.^34^ showed that at urban subsystem level, the collection system with the least impact (following a life cycle assessment) is multi-container collection between three options (the mobile pneumatic, the multi-container and the door-to-door). Nevertheless, other works in Historical Centres in Spain showed that when the organic fraction is collected separately, the pneumatic collection could be a suitable alternative because the energy requirements are balanced with the savings from the anaerobic digestion process^35^.

Citizens identify dirty food packaging with the fraction of biowaste and, therefore, for future research, citizens should be informed that these materials must be cleaned and placed in the appropriate container. Dirty paper, which is also identified as biowaste, was also found in this fraction but this is not a problem since it is biodegradable. Finally, very little glass was found, except in Zone 1. As Zone 1 is a restaurant and commercial area, wine bottles appeared in some characterizations.

If the composition of the biowaste container is compared with that of mixed waste (Table 4), a clear difference is observed in all the fractions, mainly in the biowaste with a notable increase, which is why the separate collection system has been successful. Something similar happened in similar experiments carried out in a Mediterranean area such as the one mentioned above (Catalonia), where the percentage of people participating in source separation systems has increased considerably, which was due to the incentives from the local government to improve the quality of the biodegradable fraction of municipal solid waste^32^. However, the level of separate collection of biological waste in the EU countries is very different. In countries such as Austria, Flanders (Belgium), Germany, the Netherlands, Norway, Sweden and Switzerland, the separate collection of biowaste has been in place for more than 15 years. Other countries such as Estonia, Finland, France, Ireland, Italy, Slovenia and the United Kingdom have been gradually implementing these systems over the last 15 years, while Bulgaria, Cyprus, Croatia, the Czech Republic, Greece, Hungary, Latvia, Lithuania, Malta, Poland, Portugal, Romania, Slovakia and Spain are still applying separation (albeit unevenly) in their regions (ECN, 2020).

**Table 4:** Biowaste container composition (%).

	Zone 1		Zone 2		Zone 3	
	Mean	St. dev	Mean	St. dev	Mean	St. dev
Biowaste	79.75	11.30	82.74	5.57	81.18	8.78
Inappropriate	20.25	11.30	17.25	5.52	18.82	8.78
Metal packaging	0.37	0.33	0.21	0.08	0.16	0.11
Other metals	0.01	0.02	0.01	0.02	0.03	0.04
Clean paper/cardboard	0.00	0.00	0.09	0.13	0.07	0.09
Dirty paper/cardboard	2.18	0.94	2.50	0.42	3.41	0.51
Plastic packaging	6.02	2.00	4.45	0.47	5.97	0.83
Other plastics	0.51	0.43	0.21	0.08	0.49	0.24
Sanitary cellulose	0.40	0.63	0.19	0.18	0.18	0.12
Beverages cartons	0.09	0.23	0.07	0.03	0.17	0.20
Textile	0.00	0.00	0.02	0.02	0.33	0.33
Others	0.13	0.26	0.70	0.28	0.11	0.20
Glass packaging	2.13	2.00	0.44	0.57	0.42	0.23
Fines	8.41	1.72	8.35	0.77	7.48	0.97

Table 4 shows that there are no significant differences in the biowaste percentages among the three zones, and therefore in their QCR_bw_. To determine whether it was true, it was necessary to demonstrate it statistically. For this reason, an Analysis of Variance was carried out. In this case, it was verified that normality cannot be assumed for the biowaste fraction. Consequently, the Krustal Wallis test was used with a confidence level of 95% (α = 0.05).

After comparing the means, a p-value of 0.814 (p-value > 0.05) was obtained, so it could be stated with 95% confidence that there are no statistically significant differences in the percentage of biowaste between the three areas. This fact indicates that the citizens who participated in the experience behaved in the same way in the three areas and that they all reached the same level of knowledge regarding what should be deposited in the biowaste container.

**Figure 2:**
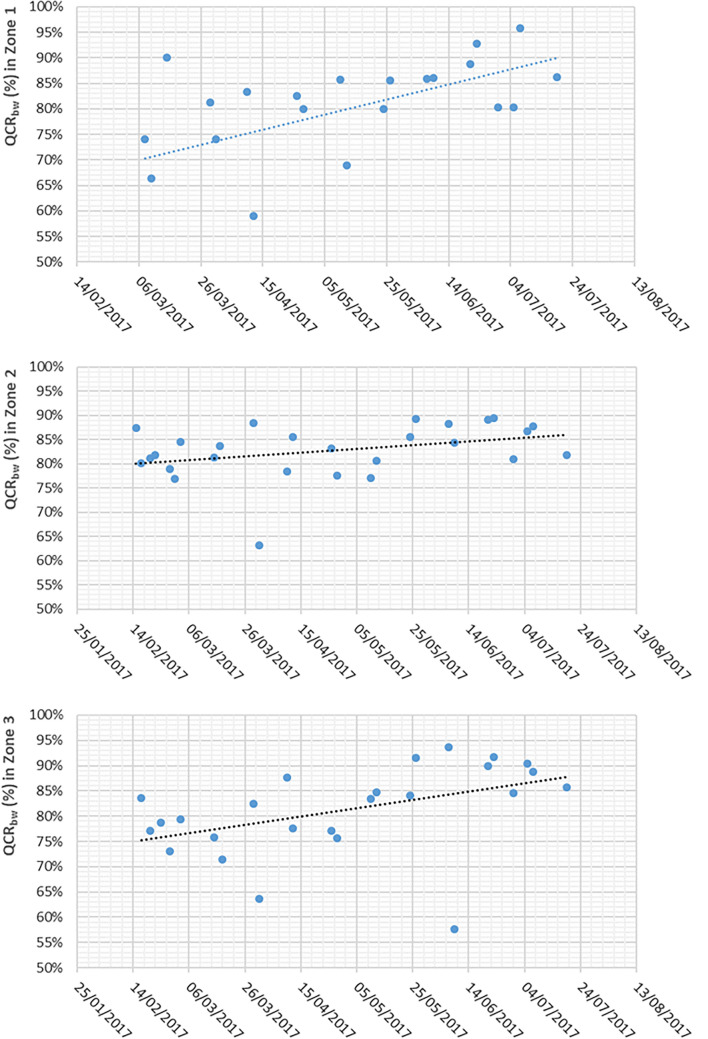
Variation of QCR_bw_ (%) over time in Zone 1, 2 and 3.

The variation in the QCR_bw_ of the biowaste container over time can be seen in Fig. 2. In all zones the QCR_bw_ increased as the pilot study progressed. Zone 1 underwent the greatest increase, going from 70% at the beginning of the experiment to 90% at the end. In Zones 2 and 3, the progress was smoother because the initial QCR_bw_ data were higher.

In the three zones, QCR_bw_ values of around 90% are reached at the end of the experiment. This increase may be mainly due to citizens’ learning and familiarization with this new separate waste collection, which implies an increase in their collaboration. Throughout the experiment there was no reinforcement of information, so it follows that citizens themselves voluntarily improved the quality of the waste in the container. Therefore, it is assumed that this is the value that can be reached after its definitive implantation in the town and that, with a continuous awareness campaign, it could be maintained over time. By the way, a pilot experience carried out in two Italian cities based on specific criteria (waste containers selections for door-to-door collection, public awareness and tariff) and solutions (door-to-door bins, warnings, criteria for historic centres) made than the level of recycling percentage increased from 47% (before) to 70% (after). More in general, in 1998 the percentage of selective collection reached 9.9%, in 2005 45.8% and in 2013 74.7%, rising to 81.1% in 2016^36^. Similar works carried out by Rada et al.^37^ showed that the adoption of enhanced collection systems and the implementation of the tariff with accurate measurement, clearly showed a way to get very high levels of waste separation.

The results presented in this work will be useful to design the future treatment line of the biowaste fraction with the aim to produce high quality compost. The QCR_bw_ results will be useful to design and choose the most suitable technology for the pretreatment stage, where the inappropriate materials are eliminated.

### The average composition of the waste in the mixed waste container

Table 5 shows the average composition of the mixed waste container for zones 1, 2, 3 and 4. The characterization was carried out in the last month of the experiment, when the QCR_bw_ of the selective collection was found to be higher.

**Table 5:** Composition of the waste in the mixed waste container.

Fraction	Mixed waste (%)							
	Zone 1		Zone 2		Zone 3		Zone 4	
	Mean	St. dev	Mean	St. dev	Mean	St. dev	Mean	St. dev
Biowaste	62.06	11.11	54.31	9.94	58.77	1.93	59.19	3.79
Inappropriate	37.94	11.11	45.69	9.94	41.23	1.93	40.81	3.79
Metal packaging	3.55	2.54	2.65	1.09	2.38	0.91	2.78	0.60
Other metals	0.20	0.28	0.30	0.52	0.38	0.62	0.29	0.10
Clean paper/cardboard	3.84	0.84	5.24	1.31	3.95	0.98	4.21	0.76
Dirty paper/cardboard	5.26	3.76	3.58	2.09	3.06	1.16	3.91	1.11
Plastic packaging	9.07	0.02	10.55	4.34	10.63	1.47	9.92	0.84
Other plastics	0.60	0.68	5.43	6.47	2.22	2.04	2.68	2.39
Sanitary cellulose	2.62	3.29	2.69	2.43	6.75	1.43	3.90	2.29
Beverages cartons	0.46	0.19	1.94	1.61	1.65	0.28	1.31	0.77
Textile	2.40	2.61	1.58	0.34	3.41	2.36	2.40	0.91
Others	3.17	2.45	7.15	6.96	3.17	1.91	4.37	2.24
Glass packaging	6.80	2.00	4.57	0.26	3.64	0.09	4.87	1.57
Fines	0.00	0.00	0.00	0.00	0.00	0.00	0.16	0.03

According to the data in Table 5, despite the fact that selective collection of light-packaging, paper/cardboard and glass is carried out in the city, significant percentages of these materials appear in the container in the four zones. It is also important to highlight that there are apparently no differences between the four zones.

Thus, to determine statistically whether there are any significant differences between the four zones regarding the percentage of biowaste in the mixed waste container, a comparison of means was conducted. For this purpose, the Kruskal Wallis test with a confidence level of 95% (α = 0.05) was used. We decided to employ this non-parametric test because the number of mixed waste composition data for each zone is small (three data).

From the results, it can be stated (with 95% confidence) that there are no significant differences in the percentages of biowaste in the mixed waste container between the four zones, since the p-value obtained is 0.7793 (p-value > 0.05). This is due to the fact that only a small part of the biowaste is diverted from the mixed waste container to separate biowaste collection, as indicated by the SR_bw_ values shown in Table 6. Therefore, the composition of the mixed waste container in Zones 1, 2 and 3 does not change significantly with respect to Zone 4.

**Table 6:** Results of the selective collection of biowaste.

Parameters	Zone 1	Zone 2	Zone 3	Total
Total gross biowaste (kg)	5,180	12,080	9,200	26,460
Total net biowaste (kg)	4,184	10,051	7,491	21,725
Biowaste generation per year (kg)	291,922	139,898	216,354	648,174
Biowaste generation in the town (%)	59.19	59.19	59.19	59.19
Collection days	137	179	179	495

				Mean
SR_bw_ (%)	1.77	8.63	4.25	4.08
NSR_bw_ (%)	1.42	7.14	3.45	3.32
QCR_bw_ (%)	79.75	82.74	81.18	81.22
DCR_gbw_ (kg/inh·day)	0.010	0.047	0.023	0.026
DCR_nbw_ (kg/inh day)	0.008	0.039	0.019	0.022
CR_bw_ (inh/cont)	99	145	374	137

### Separate collection of the biowaste and its variation over time

Table 6 shows the results of the collection in the three areas studied. The total amount of waste monitored in the pilot study was 26,460 kg, of which 21,725 kg corresponds to net biowaste (nbw). It should be noted that Zone 2 is the one where the largest amount of waste and biowaste was collected, despite being the zone with the least inhabitants. The lowest amount of waste was collected in Zone 1, but this is due to the fact that collection started 6 weeks later.

Thus, in order to compare the amount of waste collected in each area, the gross and net biowaste daily collection rates (DCR_gbw_ and DCR_nbw_) were calculated. The gross biowaste (gbw) includes the inappropriate fraction (impurities or inappropriate materials), and the net has only the biowaste. Impurities in biowaste, such as plastics, glass, metals and inert material, negatively influence the operation of anaerobic digestion plants and compost quality, and have to be removed prior to the anaerobic digestion process^29^. According to the results in Table 6, the highest values correspond to Zone 2. Zone 1 presents the worst results in DCR_gbw_ and DCR_nbw_, despite having the best CR_bw_, which may be due to the characteristics of the zone (a large number of shops, low population density and elderly population), since, the socioeconomic attributes of residents had an impact on waste separation behaviour. In other studies, carried out in the same city of Castelló, authors conducted a survey that asked citizens about their behaviour with regard to selective collection of biowaste. They confirmed that, in relation to the age, it seems that young people are more willing to pay a tax for the implementation of the biowaste collection system than older people. Regarding gender and work, men are slightly more willing to pay than women, and people that currently have a job are more willing to pay than retired people. Moreover, analysing age and education, people with primary studies are less willing to pay than people with university studies^38^. Other works showed that the educational level has the greatest influence on waste separation behaviour. Residents of different educational levels exhibited different separation behaviours, with undergraduates demonstrating more positive behaviour^39^. However, in studies carried out in China (with habits very different from those in Europe), no statistically significant difference has been found regarding the demographic characteristics such as gender, age, education, employment, and income. Unlike, citizens of different education levels have significantly different separation behaviours, with the undergraduate group demonstrating more positive behaviour than the other groups^39^.

Zone 3 has lower values than Zone 2. These two zones have similar characteristics in terms of population and endowment facilities. Therefore, the lower values are exclusively due to their higher CR_bw_, which means that citizen’s collaboration is lower, since the distance to the container is greater –a fact that was already confirmed by Gallardo et al.^27^. The data are similar to those presented in Estonia (0.109 kg/inhab·day) or Hungary (0.05 kg/inhab·day), but far from other countries such as Belgium (0.562 kg/inhab·day), Germany (0.499 kg/inhab·day), Finland (0.334 kg/inhab·day) or Italy (0.249 kg/inhab·day) (European Environment Agency, 2009).

For the calculation of SR_bw_ and NSR_bw_, data about the collection of the mixed waste fraction (fraction which includes the biowaste) for the entire city of Castelló and its composition (see Table 4, Zone 4) are available. The SR_bw_ and NSR_bw_ values appear in Table 5. These values are very low. The highest values are found in Zone 2, with a SR_bw_ of 8.63% and an NSR_bw_ of 7.14%, followed by Zone 3, with an SR_bw_ of 4.25% and an NSR_bw_ of 3.45%. The literature review and the experiences of other cities with regard to separate collection also indicate that densely populated areas with large incoming populations have difficulty achieving high separate collection rates^20^.

However, in the waste directives, the EU promulgates that only 10% of the waste will be deposited in landfills in 2030. For this reason, the waste management system, especially in some countries, should improve considerably.

Taking into account the values of SR_bw_ and NSR_bw,_ some actions can be proposed to improve them such as to increase the number of containers to collect the biowaste (decrease CR_bw_) or to collect door-to-door the biowaste of big waste producers (restaurants, hotels, schools, groceries, etc.). The impact of the weight-based tariff could improve the selective collection as Dahlén^17^ pointed in his comparison of different collection systems for sorted household waste in Sweden.

Another solution could be to separate the biowaste at the source and to collect it door-to-door. This system proved to be the best solution for the high public participation and the increase of the percentage of biowaste in the collection^40^. This decision coincides with studies conducted by Slavík et al.^41^, which fixed a series of factors that should be paramount in organizing the separate collection of biowaste, including the location of containers, and system parameters (e.g. volume of containers, frequency of collection). The results obtained from Slavík et al.^41^ confirmed how an intensive and adequate information campaign contributed to reduce the amount of improper waste in the biowaste container. Furthermore, they observed that if the location of the containers was optimized and their number increased, the public participation was greater. Therefore, the proximity of the containers to the citizens, the door-to-door collection and the supply of compostable bags to the citizens was raised as the best option from the citizen participation point of view and with the highest quality of biowaste.

From an analysis of the previous data, it can also be concluded that the collection system affects its performance, since each system defines the separation between containers differently and, therefore, its CR_bw_. Therefore, in the pilot study that was carried out, it has been possible to show that, under the same characteristics of the population, the collection system with a lower CR_bw_ has a higher SR_bw_ as Slavík et al.^41^ also demonstrated in their work.

**Figure 3:**
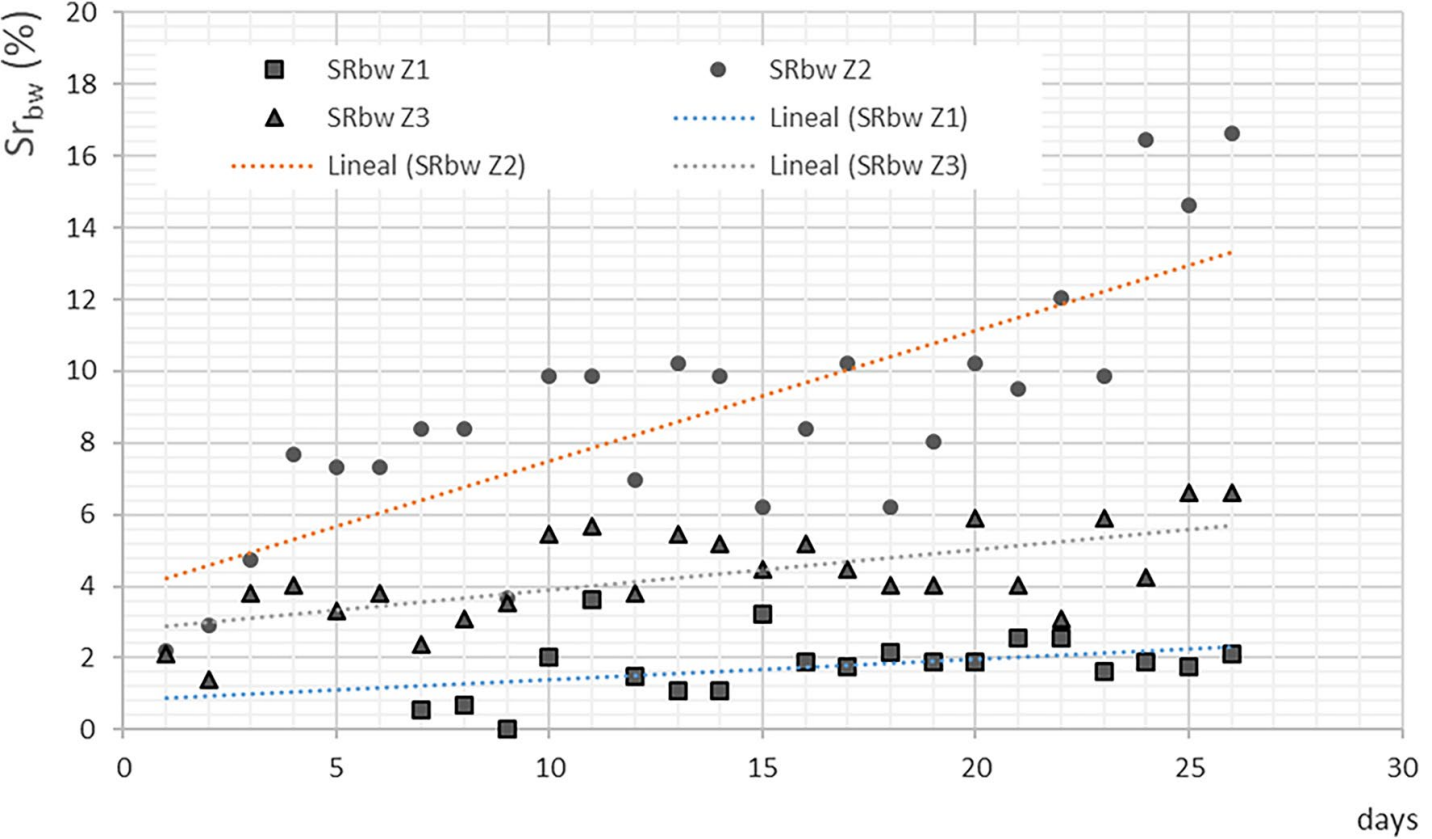
Variation in the SR_bw_ over time.

To analyse the variation in SR_bw_ throughout the experiment, the SR_bw_ values of the three zones have been represented on a graph (Fig. 3). The figure shows that in Zone 2 the SR_bw_ value increases significantly over time, in Zone 3 the increase was slighter, and in Zone 1 it increased very little. This increase is due to the higher participation, because the number of people collaborating was greater or because the participants increased their level of involvement or both.

It should be noted that the information campaign was only carried out at the beginning of the pilot study and there were no other reinforcement campaigns throughout the experiment but, even so, the data indicate that participation in the three areas increased.

Finally, the increase in the SR_bw_ and the QCR_bw_ over time makes the last month the most efficient in the experiment. Therefore, these would be the data that could be taken as achievable when implemented throughout the city. Furthermore, this awareness is in accordance with a Eurobarometer survey on “Attitudes of European citizens towards the environment”, in which it is concluded that 46% of Europeans (EU-28) considered the increasing amount of waste as one of the four biggest environmental problems in the EU. In fact, in the past six months, 66% of Europeans (EU-28) separated most of their waste for recycling (European Commission, 2020). Therefore, moving towards a circular economy requires active public participation in waste management and pre-sorting of wastes at home. In the process of establishing and improving well-performing municipal solid waste management systems the understanding of fundamental social factors to influence household behaviour is commonly underestimated but it is highly important^42^. Therefore, to increase the amount of separate collection of biowaste through a survey about the implementation of pilot projects for the separate collection of biowaste, and awareness and information campaigns aimed at emphasizing the role that consumers play in the separation at source of biowaste^38^ is essential. In fact, the circular economy could represent the answer to improve current solid waste management activities worldwide, since denote the principle of waste valorization and recycling for boosting developing economies^43^.

## Conclusions

This study presents a methodology to determine the degree of efficiency of the biowaste collection system. The results achieved are the first step towards improving a new biowaste collection system.

The study analyses the particular case of a pilot study of the selective collection of the biowaste in Castelló de la Plana (Spain). The pilot scheme focuses on the current fraction of mixed waste. From now on citizens should separate biowaste (brown container) and mixed waste (green container) at source.

Regarding QCR_bw_ the study allowed the following issues to be determined:

- In the three areas, the QCR_bw_ obtained is the same from the statistical point of view. Therefore, it has been shown that the citizens in the three areas separated their waste at source in a similar way.
- The percentage of inappropriate material is 20%, consisting mainly of recyclable materials.
- It has been verified that in the three zones the QCR_bw_ increased as the pilot study progressed, finally reaching 90%. This increase may be due to citizen learning and familiarization with this new selective waste collection. Therefore, it is hoped that this is the value that can be reached after it is implemented definitively in the city.
- Regarding the mixed waste container, the four zones present QCR_bw_ values that are not statistically different. This fact shows that there has been no significant transfer of biowaste to the brown container.

To increase QCR_bw_, citizens need more information about how to manage food-containing packaging. Other measure could be to sell compostable bags at reasonable prices or they can even be distributed for free. The results of this work will be useful to design a future treatment line for the biowaste collected separately.

On the other hand, regarding the amounts of waste collected separately in the brown container, this study allowed the following issues to be determined:

- Zone 1presents the worst results in DCR_gbw_ and DCR_nbw_, despite having the best CR_bw_. Its characteristics are different from the ones of the other zones and this fact could have affected the results.
- On analysing the DCR_gbw_ and DCR_nbw_ from Zones 2 and 3 it has been shown that their value increases when CR_bw_ decreases and, therefore, to have better results it will be necessary to decrease the CR_bw_, that is, to reduce the distance from the citizen to the container.
- The SR_bw_ increases in the three zones over time, although in different ways, which means that citizens’ collaboration has increased.
- The collection system affects the SR_bw_, since each system defines the separation between containers differently and, therefore, its CR_bw_ will be different.

The increase in SR_bw_ and QCR_bw_ over time makes the last month of the experiment the most efficient. Thus, it can be said that there is a positive evolution of the experiment, which encourages implementation of the system throughout the city.

To rise SR_bw_ considerably, some measures are proposed such as to increase the number of containers (lower CR_bw_), to collect the biowaste from big producers separately, to collect the waste door-to-door in some zones of the town, to design information and awareness campaigns and to deepen the concept of weight-based tariff to apply it in the future. All the measures derived from this work will contribute to promote the circular economy from the waste collection point of view as the biowaste collected will have a greater quality to be turned into compost.

Finally, the proposed methodology and its results in the pilot study can be useful at the international level when implementing a separate biowaste collection system in a city with similar characteristics.

## Data Availability

Not applicable; All the data with which this article has been prepared is included in the article itself.

## Abbreviations

bw: Biowaste
CR: Containerization rate
DCR: Daily collection rate
EU: European Union
gbw: Gross biowaste
mx: Mixed waste
nbw: Net biowaste
NSR: Net separation rate
QCR: Quality in container rate
SR: Separation rate
st. dev.: Standard deviation
VC: Variation coefficient
